# Supplementary material for: Helical reconstruction in RELION
Source: J Struct Biol. 2017 Jun;198(3):163–76. doi: 10.1016/j.jsb.2017.02.003 (PMC5479445; doi:10.1016/j.jsb.2017.02.003)
Supplement: Supplementary data [file mmc1.pdf]

# Supplementary material to:

## Helical reconstruction in RELION

Shaoda He & Sjors H.W. Scheres*

MRC Laboratory of Molecular Biology, Francis Crick Avenue, Cambridge Biomedical Campus, CB2 0QH, Cambridge, UK

* Correspondence to: scheres@mrc-lmb.cam.ac.uk

### *Contents*

**Supplementary Figure 1:** Helical lattice determination

**Supplementary Figure 2:** Convergence of helical parameters within 3D auto-refinement runs

**Supplementary Figure 3:** Refinements with varying inter-box distances

**Supplementary Figure 4:** Prevention of overfitting

**Supplementary Figure 5:** Comparison of different priors

**Supplementary Table 1:** Comparison of manual and template-based particle picking

**Supplementary Table 2:** Local optimisation of helical parameters

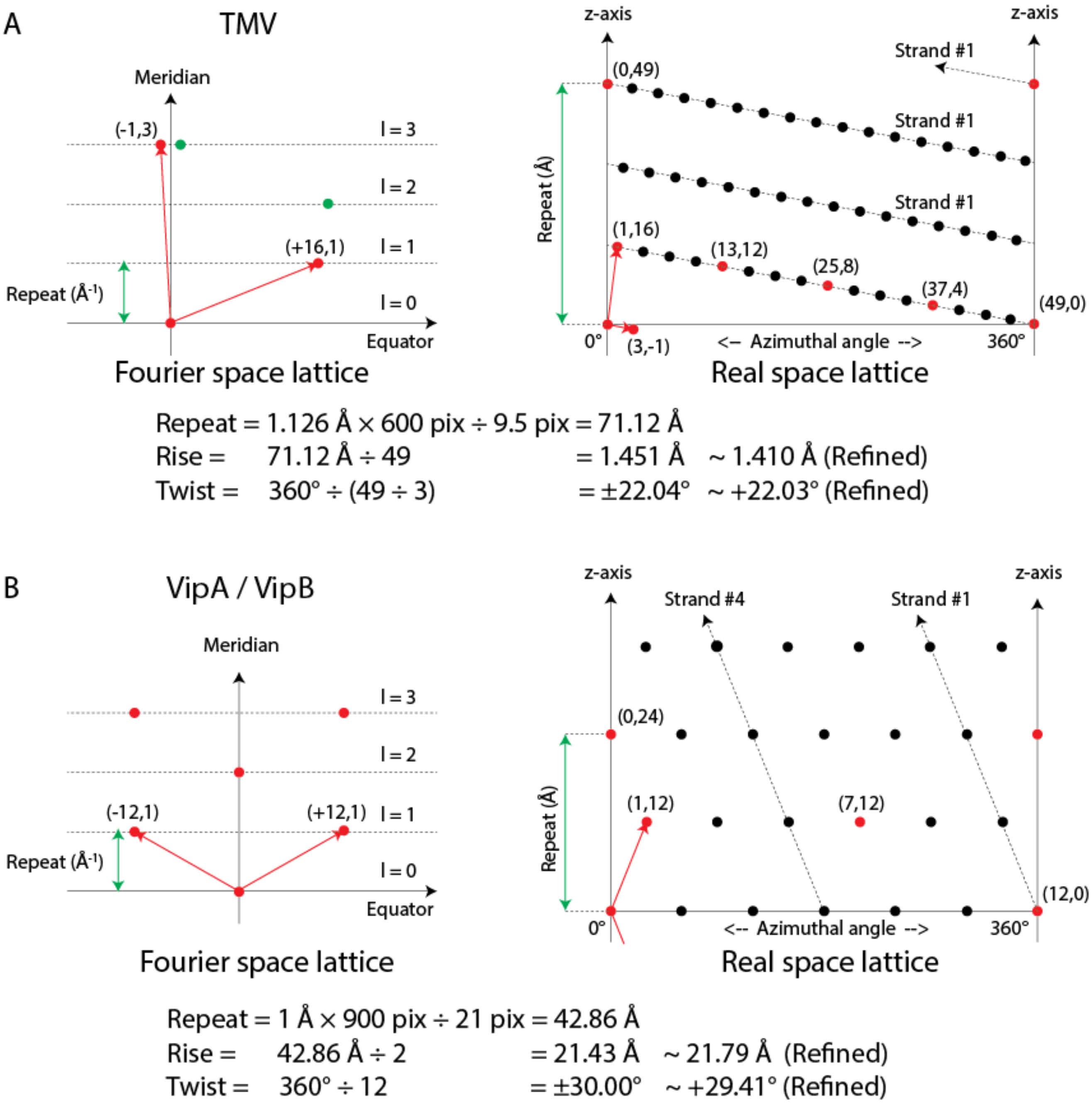


**Supplementary Figure 1:** Helical lattice determination for TMV (**A**) and VipA/VipB (**B**). The Fourier-space lattice (red arrows, left) is converted into a real-space lattice (red lines, right) by a 90° rotation and a mirror operation. The reciprocal repeat distance (green arrow, left) is the distance in real-space where the lattice folds back on itself on the z-axis (green arrow, right). The helical rise is then calculated by dividing the repeat distance by the number of lattice points in a full repeat (49 for TMV, 2 for VipA/VipB). The helical twist is calculated by dividing 360° by the number of lattice points in a single turn.

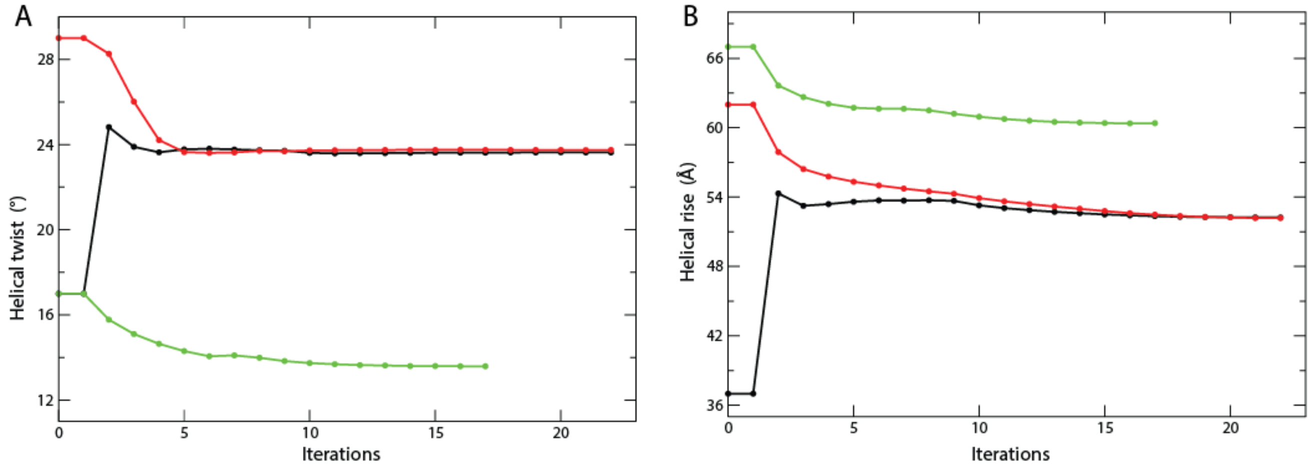


**Supplementary Figure 2:** Convergence of helical parameters within 3D auto-refinement runs. Plots of the refined helical twist (A) and rise (B) for each iteration of three different 3D auto-refinement runs: starting from initial twist and rise settings of: 17° and 37Å (black); 29° and 62Å (red); and 17° and 67Å (green). The first two refinements converged onto the correct values, whereas the latter did not.

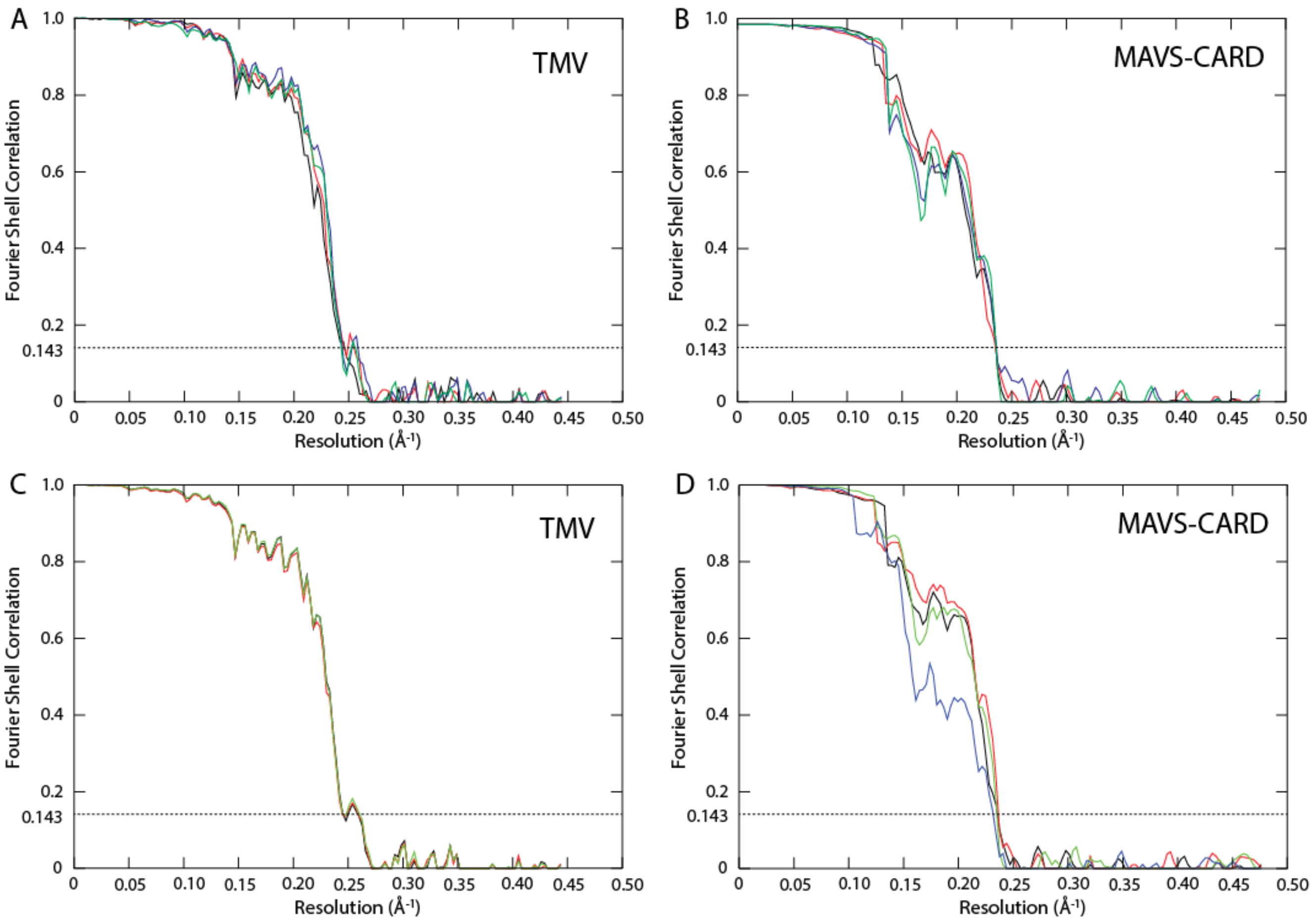


**Supplementary Figure 3:** Refinements with varying inter-box distances. **A.** FSC curves for TMV refinements with inter-box distances corresponding to 5 (black), 15 (red) and 30 (blue) and 75 (green) new asymmetrical units per segment, corresponding to 2.1 %, 6.3%, 12.5% and 31.3% of the box size, respectively. The rise of TMV is 1.41 Å and the box size is 338 Å. The mask diameter in these refinements was fixed at 90% of the box size. **B.** FSC curves for MAVS/CARD refinements with inter-box distances corresponding to 2 (black), 8 (red), 14 (blue), and 20 (green) new asymmetrical units per segment, corresponding to 3.2%, 12.9%, 22.5% and 32.2% of the box size, respectively. The rise of MAVS/CARD is 5.07Å and the box size is 315 Å. The mask diameter in these refinements was fixed at 90% of the box size. **C.** FSC curves for TMV refinements with mask diameters corresponding to 90% (black), 85% (red) and 80% (green) of the box size. The inter-box distance in these refinements was fixed to yield 30 new asymmetrical units per segment. **D.** FSC curves for MACS/CARD refinements with mask diameters corresponding to 90% (black), 80% (red), 70% (green) and 60% (blue) of the box size. The inter-box distance in these refinements was fixed to yield 8 new asymmetrical units per segment.

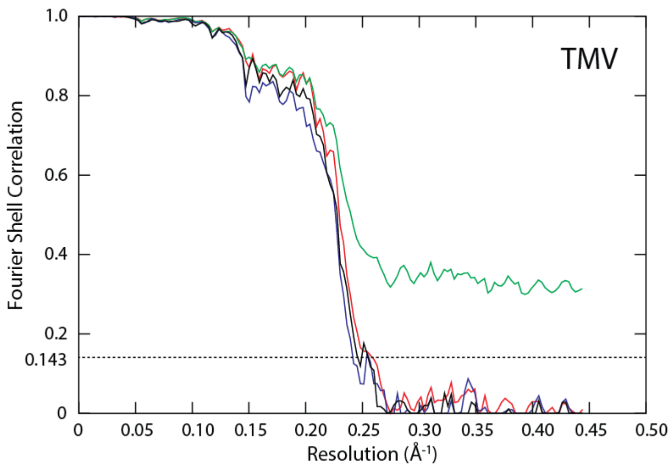


**Supplementary Figure 4:** Prevention of overfitting. The FSC curve for TMV refinements with: filament-based half-sets with a top-hat prior on the translation along the helical axis (black); filament-based half-sets without a translational prior (blue); segment-based half-sets with a translational prior (red); and segment-based half-sets without a translational prior (green).

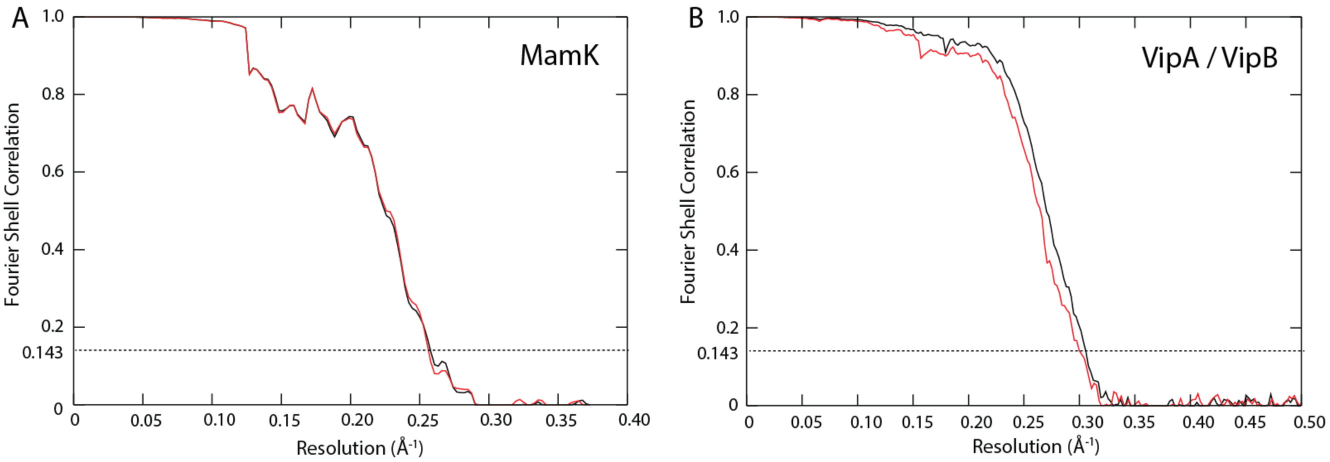


**Supplementary Figure 5:** Comparison of different priors. FSC curves of post-processed maps for MamK (**A**) and VipA/VipB (**B**), using either Gaussian priors (black) or top-hat priors (red) in the refinement.

**Supplementary Table 1:** Comparison of manual picking and template-based auto-picking for the VipA/VipB and the MAVS/CARD filaments. The number of segments after picking and after selection of good classes from a single 2D classification run are shown, together with the resolution of the map after 3D auto-refinement.

| | **Segments picked** | **Segments kept** | **Resolution** |
|---|---|---|---|
| **VipA/VipB (manual)** | 10,830 | 10,211 | 3.3 Å |
| **VipA/VipB (auto)** | 11,114 | 9,480 | 3.3 Å |
| **MAVS/CARD (manual)** | 34,397 | 34,397 | 4.3 Å |
| **MAVS/CARD (auto)** | 227,176 | 133,237 | 4.1 Å |

**Supplementary Table 2:** Local optimisation of helical parameters for the MamK data set. Starting from different initial estimates for the helical twist (top row, with search ranges between square brackets) and rise (first column, with search ranges between square brackets), the final refined values for the helical twist (top), rise (middle) and resolution (bottom) are shown. Asterisks and red colours are used to indicate values that deviate more than 1° or 1 Å from the correct ones, which are shown in bold and green in the middle.

| **Twist →**<br>**Rise ↓** | **17°**<br>**[14-26°]** | **19°**<br>**[16-26°]** | **21°**<br>**[18-26°]** | **23.746°**<br>**[21-26°]** | **25°**<br>**[21-29°]** | **27°**<br>**[21-31°]** | **29°**<br>**[21-33°]** |
|---|---|---|---|---|---|---|---|
| **37 Å**<br>**[32-57 Å]** | 23.63°<br>52.24Å<br>6.47Å * | 23.73,<br>52.13Å<br>5.44 Å | 23.72,<br>52.14Å<br>5.44Å | 23.58,<br>52.28Å<br>6.7Å * | 24.23°<br>52.14Å<br>6.05Å * | 23.60,<br>52.16Å<br>5.44Å | 28.54 *<br>37.03Å *<br>8.93Å * |
| **42 Å**<br>**[37-57 Å]** | 23.18°<br>52.26Å<br>6.36Å * | 23.73°<br>52.11Å<br>4.69Å | 23.64°<br>52.09Å<br>4.75Å | 23.74°<br>52.12Å<br>5.36Å | 20.97° *<br>46.09Å *<br>6.70Å * | 23.69°<br>52.13Å<br>4.81Å | 31.96° *<br>51.02Å *<br>8.53Å * |
| **47 Å**<br>**[42-57 Å]** | 23.71°<br>52.10Å<br>4.75Å | 23.73°<br>52.10Å<br>4.81Å | 23.72°<br>52.10Å<br>4.63Å | 23.73°,<br>52.12Å<br>4.63Å | 23.73°<br>52.10Å<br>4.63Å | 23.73°<br>52.11Å<br>4.75Å | 32.31° *<br>52.26Å *<br>7.36Å * |
| **52.15 Å**<br>**[47-57 Å]** | 23.65°<br>52.19Å<br>6.25Å * | 23.73°<br>52.11Å<br>4.81Å | 23.76°,<br>52.12Å,<br>4.63Å | **23.77°,**<br>**52.12Å,**<br>**4.69Å** | 23.74°<br>52.12Å<br>4.63Å | 23.73°<br>52.12Å<br>4.63Å | 23.71°<br>52.13Å<br>4.63Å |
| **57 Å**<br>**[47-62 Å]** | 23.69°<br>52.13Å<br>4.75Å | 23.72°<br>52.11Å<br>4.69Å | 23.73°<br>52.16Å<br>4.75Å | 23.75°<br>52.12Å<br>4.63Å | 23.74°<br>52.12Å<br>4.69Å | 23.71°<br>52.12Å<br>4.63Å | 23.73°<br>52.12Å<br>4.63Å |
| **62 Å**<br>**[47-67 Å]** | 16.00° *<br>53.14Å<br>7.36Å * | 23.73°<br>52.18Å<br>4.87Å | 23.69°<br>52.61Å<br>6.15Å | 23.73°<br>52.13Å<br>4.63Å | 23.73°<br>52.12Å<br>4.81Å | 23.76°<br>52.14Å<br>4.75Å | 23.74°<br>52.18Å<br>5.36Å |
| **67 Å**<br>**[47-72 Å]** | 13.59° *<br>60.39Å *<br>7.98Å * | 18.29° *<br>59.40Å *<br>8.34Å * | 22.82°<br>53.91Å<br>7.66Å * | 23.14°<br>53.34Å<br>6.82Å * | 22.98°<br>55.35Å *<br>7.66Å * | 24.78° *<br>63.00Å *<br>8.34Å * | 31.24° *<br>62.99Å *<br>7.98Å * |
